# Long-term Fertilization Structures Bacterial and Archaeal Communities along Soil Depth Gradient in a Paddy Soil

**DOI:** 10.3389/fmicb.2017.01516

**Published:** 2017-08-15

**Authors:** Yunfu Gu, Yingyan Wang, Sheng’e Lu, Quanju Xiang, Xiumei Yu, Ke Zhao, Likou Zou, Qiang Chen, Shihua Tu, Xiaoping Zhang

**Affiliations:** ^1^Department of Microbiology, College of Resource Science and Technology, Sichuan Agricultural University Chengdu, China; ^2^Soil and Fertilizer Institute, Sichuan Academy of Agricultural Sciences Chengdu, China

**Keywords:** soil profile, miseq sequencing, soil bacteria, soil archaea, specific taxa, network analysis, 16S rRNA gene amplicon

## Abstract

Soil microbes provide important ecosystem services. Though the effects of changes in nutrient availability due to fertilization on the soil microbial communities in the topsoil (tilled layer, 0–20 cm) have been extensively explored, the effects on communities and their associations with soil nutrients in the subsoil (below 20 cm) which is rarely impacted by tillage are still unclear. 16S rRNA gene amplicon sequencing was used to investigate bacterial and archaeal communities in a Pup-Calric-Entisol soil treated for 32 years with chemical fertilizer (CF) and CF combined with farmyard manure (CFM), and to reveal links between soil properties and specific bacterial and archaeal taxa in both the top- and subsoil. The results showed that both CF and CFM treatments increased soil organic carbon (SOC), soil moisture (MO) and total nitrogen (TN) while decreased the nitrate^_^N content through the profile. Fertilizer applications also increased Olsen phosphorus (OP) content in most soil layers. Microbial communities in the topsoil were significantly different from those in subsoil. Compared to the CF treatment, taxa such as *Nitrososphaera*, *Nitrospira*, and several members of *Acidobacteria* in topsoil and Subdivision 3 *genera incertae sedis*, *Leptolinea*, and *Bellilinea* in subsoil were substantially more abundant in CFM. A co-occurrence based network analysis demonstrated that SOC and OP were the most important soil parameters that positively correlated with specific bacterial and archaeal taxa in topsoil and subsoil, respectively. *Hydrogenophaga* was identified as the keystone genus in the topsoil, while genera *Phenylobacterium* and *Steroidobacter* were identified as the keystone taxa in subsoil. The taxa identified above are involved in the decomposition of complex organic compounds and soil carbon, nitrogen, and phosphorus transformations. This study revealed that the spatial variability of soil properties due to long-term fertilization strongly shapes the bacterial and archaeal community composition and their interactions at both high and low taxonomic levels across the whole soil profile.

## Introduction

Soil microbes play key roles in the functions of ecosystems by cycling nutrients, degrading organic material and pollutants as well as in maintaining the quality of groundwater (Madsen, 1995; Fierer et al., 2003a; King, 2014). In the topsoil (0–20 cm, tilled layer) both the microbial biomass and diversity are the greatest, yet in the subsoil (below 20 cm) microbes are also diverse and abundant due to the large volume of soil on a depth-weighted basis (Will et al., 2010; Li et al., 2014). Soil microbial communities varied significantly along with the soil depth, and the microbial diversity of microorganisms typically decreases with depth (Li et al., 2014; He et al., 2017). Fertilization, an essential agricultural practice used primarily to increase nutrient availability to crop plants, causes concomitant changes in the soil properties and microbial communities (Marschner et al., 2003). Studies have reported that over fertilization with nitrogen can result in negative consequences to the environment such as soil acidification, decreased soil microbial activity, enhanced nitrification and the leaching of nitrate nitrogen (Nowinski et al., 2008; Guo et al., 2010; Ramirez et al., 2010). In contrast, the combined use of inorganic and organic fertilizer likely increase the microbial diversity, biomass and activity, and helps to maintain even increase the soil nutrients (Birkhofer et al., 2008; Kumar et al., 2017). Nutrients added to the soil by fertilizer may directly alter the abundance and composition of microbial community in topsoil; in subsoil soluble nutrients leaked from topsoil change these microbial parameters indirectly (Stowe et al., 2010; Eilers et al., 2012). However, little is known about the characterization and spatial variability of microbial communities with depth in fertilized paddy soils (Bao et al., 2015; Yu et al., 2015).

Certain bacterial and archaeal taxa at high taxonomic levels (e.g., phylum or class) have shown ecological coherence since they respond predictably to environmental variables (Philippot et al., 2010; Cederlund et al., 2014). The abundance of *Proteobacteria* and *Actinobacteria* in the topsoil increased under long-term combined use of chemical and organic fertilizer while the abundance of *Acidobacteria* decreased (Li et al., 2014). However, little is known about the response of specific soil microbial taxa at low taxonomic levels (e.g., genus or species) to long-term fertilization. Long-term application of organic fertilization seems to select for certain microbial taxa at low taxonomic levels that feed primarily on organic substrates and proliferate greatly, resulting in the changes in microbial community composition and soil nutrient status (Cederlund et al., 2014; Li et al., 2014, 2017). As a result, specific microbial taxa of which the abundances are substantially increased by long-term fertilization should show some degree of connections with soil nutrients. These predictable responses of specific microbial taxa make them possibly useful indicators of soil nutrient status and sustainability (Smit et al., 2001; Hartman et al., 2008). Moreover, microbes including these specific microbial taxa formed complex interaction webs in soil ecosystem, and understanding the interactions between them is critical to explore the complexity of functional processes (Faust and Raes, 2012). Co-occurrence network analysis, based on the analysis of correlations between the abundances of microbial taxa, has provided comprehensive perspective into the microbial association patterns and the ecological functions guiding community assembly, such as commensalism, competition, and predation. Thus, network analysis has been used to describe microbial co-occurrence relations in diverse environments including marine sediments, soil, and waste water. However, knowledge on the effects of long-term fertilization on the networks of taxon co-occurrence of microbial communities across the whole soil profiles is still scarce.

To address these questions, we analyzed microbial communities in a long-term fertilization field experiment site in Sichuan, China. Our previous studies on this calcareous purplish paddy soil site showed that the NPK and NPK combined with farmyard manure (NPKM) treatments significantly increased the rice and wheat yields, decreased the soil pH and modified the microbial community in the topsoil (Gu et al., 2008). In this study, two typical fertilizer treatments [chemical fertilizer (CF; NPK), and chemical fertilizer combined with farmyard manure (CFM; NPKM)] were compared with a non-fertilized treatment (CK), and deep 16S rRNA gene amplicon sequencing was used to examine the changes in bacterial and archaeal communities with depth. Differential abundance analysis and co-occurrence based network analysis were performed to unravel the potential effects of specific bacterial and archaeal taxa and their associations with soil nutrients. Specifically, we examined (1) the responses of microbial communities to long-term fertilizer applications across the soil profile, (2) which specific taxa in different soil depths are substantially stimulated by long-term fertilization, and (3) the associations of specific bacterial and archaeal taxa responding to long-term fertilization in the topsoil and subsoil. We hypothesized that (i) the topsoil, which is directly affected by long-term fertilization, would host a distinct composition and diversity of soil microbial communities compared to those in subsoil; and (ii) specific taxa involved in nutrient cycling would be substantially stimulated by long-term NPKM fertilization throughout the depth of soil profile due to the incorporation of farmyard manure. Moreover, we hypothesized that (iii) specific taxa of which the abundances are substantially changed by long-term fertilization would form distinct association patterns in the topsoil and subsoil.

## Materials and Methods

### Field Site and Experimental Design

The experimental site was a ‘N, P, K long-term fertilization field experiment’ site on a calcareous purplish paddy field established in 1982 in Chuanshan (30°10′50″N, 105°03′26″E), Sichuan, China. The site which has an annual average temperature of 17.4°C and mean annual precipitation of 930 mm was described in a previous study (Gu et al., 2008). The experiment included three treatments, CF (NPK), chemical fertilizer CFM (NPKM) and unfertilized control (CK), in three replicate 13.2 m^2^ plots. In the fertilizer treatments, the application rates of inorganic fertilizers were similar to local traditional application rates: N, 120 kg ha^-1^; P_2_O_5_, 60 kg ha^-1^; K_2_O, 60 kg ha^-1^. CFM treatment received 3 × 10^4^ kg ha^-1^ pig manure. The fertilizer treatments remained the same each year. All P as superphosphate and K as K_2_SO_4_ were applied as basal fertilizers, while N as urea was split into 70% as basal application and 30% as topdressing for rice at tillering. Soil original physico-chemical properties prior to the experiment are in Gu et al. (2008). The mean grain yields in the CK, CF, and CFM treatments were 2967.7 kg ha^-1^, 7116.8 kg ha^-1^, and 7496.5 kg ha^-1^, which were significantly different (*P* < 0.05) (Gu et al., 2008).

### Soil Sampling and Selected Soil Properties Measurement

In 2012, after the summer rice was transplanted and all the plots were flooded for about 55 days, five sampling points were chosen randomly in each plot and combined as one composite sample. Based on the soil profile, soil samples were collected at the following depth intervals (cm): 0–20, 20–40, 40–60, and 60–90 cm. Finally, a total of 36 soil samples were taken from the experiment site. After the removal of visible roots and fresh litter material, the composite samples were homogenized and then separated into two parts: approximately 50 g was packed into a sterile bag, immersed in liquid nitrogen instantly and stored at -70°C for DNA analysis, while the other part (approximately 800 g) was air-dried at ambient room temperature (∼25°C) and sieved through a 6-mm sieve for soil physicochemical parameter determination.

Soil pH was measured with a compound electrode (E-201-C, Shanghai Shengguang Instrument Co. Ltd. Shanghai, China) using a soil-to-water ratio of 1:1. Soil moisture (gravimetric water content) was measured using a wet/dry soil conversion with a soil subsample dried at 105°C for 12 h. Soil organic carbon (SOC) and total N (TN) were determined by dichromate oxidization and Kjeldahl digestion, respectively. Ammonium–N (NH_4_^+^–N) and nitrate–N (NO_3_^-_^N) were extracted by shaking 5 g of field-moist soil with 50 mL of 0.01 M CaCl_2_ for 30 min. Filtered extracts were kept at -20°C until NH_4_^+^–N and NO_3_^-_^N concentration were measured in an AA3 flow injection analyzer (FIA SFA CFA, Germany). Soil Olsen phosphorus (OP) and available potassium (AK) were extracted with sodium bicarbonate and ammonium acetate (Lu, 1999), respectively.

### DNA Extraction

Soil crude DNA was extracted from 0.5 g fresh soil with three replications using a FastDNA spin kit for soil (Qbiogene, Carlsbad, CA, United States) by following the manufacturer manual. DNA quality was assessed based on the spectrometry absorbance ratios at wavelengths of 260/280 nm and 260/230 nm by a NanoDrop ND-1000 Spectrophotometer (NanoDrop Technologies Inc., Wilmington, DE, United States). The integrity of the DNA extracts was confirmed by electrophoresis. DNA samples were stored at -20°C.

### Amplification of 16S rRNA Gene

Sequencing of PCR amplicons of 16S rRNA gene was conducted with the Illumina MiSeq (Illumina, San Diego, CA, United States) targeting the V4 hyper variable regions (515F, 5′-GTGCCAGCMGCC-GCGGTAA-3′ and 806R, 5′-GGACTACHVGGGTWTCTAAT-3′) for 2 × 150-bp paired-end sequencing (Caporaso et al., 2012). PCR reactions (Qiagen, Valencia, CA, United States) were performed in a 50 μl mixture containing 0.25 μl of TaKaRa Ex Taq HS at 5 U/μl, 4 μl of dNTP Mixture (2.5 mM each), 5 μl of 10 × Ex Taq Buffer (Mg^2+^ Plus), 1 μl of each primer at 10 mM and 1 μl of 10-fold diluted template DNA. The thermal program was as follows: initial denaturation at 94°C for 4 min, followed by 30 cycles of 94°C for 20 s, 53°C for 25 s, 68°C for 45 s, and a final extension step of 10 min at 68°C. PCR products (3 μl) were examined by agarose gel electrophoresis, and then 5 μl of triplicate reactions were mixed together and quantified with PicoGreen (Invitrogen Ltd., Paisley, United Kingdom). Approximately 200 ng PCR products from each sample were pooled together and purified with a QIAquick PCR Purification Kit (QIAGEN), and then re-quantified with PicoGreen. Denaturation was performed by mixing 10 μl of combined PCR products (2 nM) and 10 μl 0.1 N NaOH. Denatured DNA was diluted to 6 pM and mixed with an equal volume of 6 pM PhiX library. Finally, the 600 μl mixture was loaded into the reagent cartridge and run on a MiSeq sequencer (Illumina) for 300 cycles.

### Illumina Data Analysis

All reads were assembled and quality-filtered using a fast length adjustment of short reads (FLASH) software (Magoc and Salzberg, 2011) and QIIME pipeline (Caporaso et al., 2010), respectively. Only those reads with phred-quality score more than 20 and length over 300 bps were considered for further analysis. Those reads containing ambiguous alphabets, harboring two or more mismatches in primer or unable to be assembled were discarded during quality polishing. Operational taxonomic units (OTUs) were picked using the UPARSE pipeline (Edgar, 2013) at 97% identity. Chimeric sequences were removed using UCHIME (Caporaso et al., 2012). The RDP classifier was used to pick representative sequences for the OTUs and to assign taxonomic data to each representative sequence at the 70% threshold (Caporaso et al., 2010). Sequences which could not be assigned were removed. The sequence data were submitted to NCBI Sequence Read Archive^1^ with accession number SRS2127454. After OTU clustering at 97% sequence identity, removal of singletons and resample at 9500 sequences per sample, 34030 OTUs for all the 36 soil samples were obtained and used in downstream analysis. The Good’s coverage and rarefaction curves were calculated using QIIME pipeline and ‘rarecurve’ function in R package vegan, respectively (Caporaso et al., 2010; R Development Core Team, 2010). The Good’s coverage was ranged from 74.2 to 87.4% with an average of 80.2% (Supplementary Table S1). Meanwhile, the rarefaction curves did not reach a plateau, indicating that further sequencing could have revealed additional species coverage (Supplementary Figure S1).

### Statistical Analysis

Normality and variance homogeneity of community data were analyzed using Shapiro–Wilks and Levene’s test, respectively. Principal coordinates analysis (PCoA) was applied to estimate the microbial community structure using Bray–Curtis dissimilarities based on “Hellinger” transformed community data (Legendre and Gallagher, 2001). Analysis of similarity (ANOSIM) was used to test the dissimilarity between microbial community structure in topsoil (0–20 cm) and in subsoil (20–90 cm) (Zhou et al., 2012). After the soil edaphic parameters were standardized with the “decostand” function, distance-based redundancy analysis (db-RDA) was performed to correlate these parameters to microbial community structure in both topsoil and subsoil using “capscale” function in R package vegan (R Development Core Team, 2010). The significance of db-RDA models was tested using “permutest” function in vegan with 999 permutations. The goodness-of-fit (R2) and associated statistical significance (*P*-value) of each edaphic factor in db-RDA model were verified using “envfit” function in vegan.

Differential abundance analysis was performed in R package DESeq2 (Love et al., 2014). *P*-values were adjusted for multiple testing with the procedure described by Benjamini and Hochberg (1995), and a false discovery rate (FDR) of 10% was selected to denote statistical significance (Love et al., 2014; Whitman et al., 2016). Enriched and depleted OTUs were defined using the methods described by Li et al. (2017).

### Co-abundance Network Analysis

To minimize pairwise comparison and network complexity, only OTUs with large differential abundance (log_2_ fold change >|±1| and FDR-adjusted *P*-value < 0.1) were selected for network analysis (Li et al., 2017). The interaction network was inferred based on Spearman rank correlation matrix constructed with Python package scipy (Oksanen et al., 2013). All *P*-values were adjusted using Benjamini and Hochberg FDR controlling procedure (Benjamini et al., 2006) in R package multtest (Pollard et al., 2013). Connections between identical nodes were removed before network construction using Python package igraph (Csardi and Nepusz, 2006). Based on correlation coefficient thresholds and corresponding cutoffs of FDR-adjusted *P*-values for correlation, two interaction networks were inferred using differentially abundant OTUs from 0 to 20 cm topsoil (38 OTUs) and 20–90 cm subsoil (107 OTUs). The cutoffs of correlation coefficient were automatically determined as ±0.79 for topsoil and ±0.69 for subsoil through random theory-based methods (Luo et al., 2006). The topological features of the abundance correlation networks were not compared in this study because of the different correlation coefficient cutoffs used in meta-network construction. The threshold of FDR-adjusted *P*-values was 0.001. Network properties were calculated using Python package igraph (Csardi and Nepusz, 2006). Node level topological features including degree and betweenness centrality were calculated to identify potential keystone species. The network modules were detected using greedy modularity optimization algorithm (Newman, 2006). Modules with four or more nodes were selected for further analysis. The sources of nodes in each module were determined using an in-house Python script based on the results of differential abundance analysis. To identify the primary driving force of each network module, mantel test was performed between nodes in each network module and associated environmental factors. Networks were visualized using Cytoscape v.3.2.1 (Shannon et al., 2003).

## Results

### Soil Physico-Chemical Parameters

**FIGURE 1:**
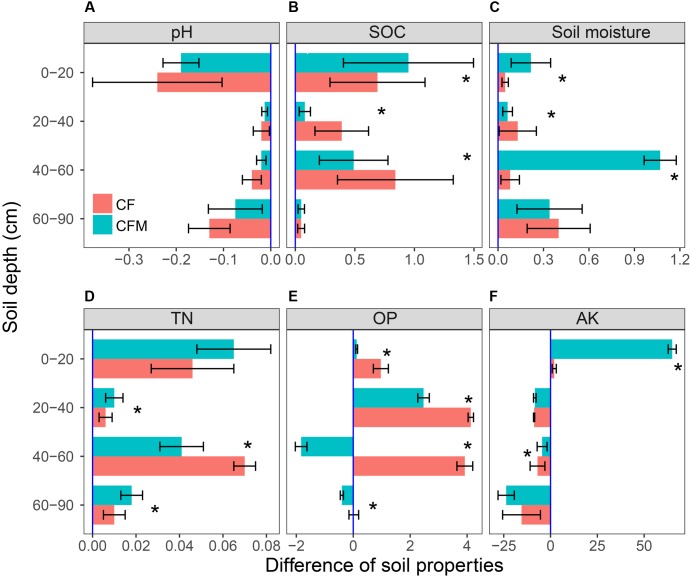
Soil properties at 0–90 cm depths under long-term applications (32 years) of NPK fertilizer (CF) and NPK combined with farmyard manure (CFM) compared with the non-fertilized control (CK). SOC, soil organic carbon; Soil moisture: gravimetric water content; TN, total nitrogen; OP, Olsen phosphorus (available phosphorus); and AK, available potassium. Error bars represent the standard error of the mean (*n* = 3). ^∗^ means significantly difference among three different long-term fertilization treatments at the level of *P* = 0.05.

Compared to the unfertilized control (CK), the two fertilizer treatments led to lower pH and higher soil moisture, SOC, TN, OP, and AK content in topsoil (0–20 cm) (**Figure 1**). Compared to the topsoil, the soil pH in both the CF and CFM treatments was higher in the subsoil (20–90 cm) (**Figure 1A**), while soil moisture, SOC, and TN were lower in both CF and CFM treatments at the subsoil (**Figures 1B–D**). In the topsoil, both soil moisture and SOC in CFM treatment was significantly higher than those in CF (*P* = 0.05) (**Figures 1B,C**). Fertilizer applications led to higher OP content in most soil layers, and significantly higher in CF treatment that those in CFM (*P* = 0.05) (**Figure 1E**). Soil AK in the topsoil was also significantly higher in the CFM (*P* = 0.05), while they were low in both CF and CFM treatments in the subsoil (**Figure 1F**). In addition, ammonium–N content was higher than nitrate^_^N content (**Table 1**). Unlike ammonium–N with non-uniform distribution within the soil profile, nitrate^_^N content significantly decreased with depth. Fertilizer applications produced significant decreases in nitrate^_^N content through the profile (**Table 1**) (*P* = 0.05).

**Table 1:** Ammonium–N (NH_4_^+^–N) and nitrate–N (NO_3_^-^–N) contents at the 0–90 cm depth under long-term fertilizer treatments.

Soil depth (cm)	NH_4_^+^–N (mg kg^-1^)			NO_3_^-^–N (mg kg^-1^)		
		
	CK	CF	CFM	CK	CF	CFM
0–20	3.93 ± 0.11a^a^C^b^	5.14 ± 0.02aB	8.29 ± 0.06aA	6.74 ± 0.15aA	2.43 ± 0.88bA	2.10 ± 0.34bA
20–40	8.71 ± 0.07aA	5.75 ± 2.00bA	7.98 ± 0.03aA	2.72 ± 0.01aB	2.12 ± 0.16aA	2.09 ± 0.12aA
40–60	7.12 ± 0.09aB	5.85 ± 0.07bA	5.39 ± 0.09bC	2.57 ± 0.25aB	1.81 ± 0.30bC	1.71 ± 0.06aA
60–90	7.61 ± 0.06aA	4.32 ± 0.08bC	6.75 ± 0.06aB	1.12 ± 0.15bC	1.19 ± 0.05aB	0.53 ± 0.13cB


*CK, no fertilizer; CF, NPK fertilizer; CFM, NPK fertilizer combined with pig manure. Values in the column are mean ± SE (stand error). ^a^Treatment means within a depth followed by the same lower case letter (a, b, and c) are not significantly different (*P* > 0.05).*

*^b^Depth means within a column followed by the same upper case letter (A, B, and C) are not significantly different (*P* > 0.05).*

### Relative Abundance of Dominant Phyla and Orders

**FIGURE 2:**
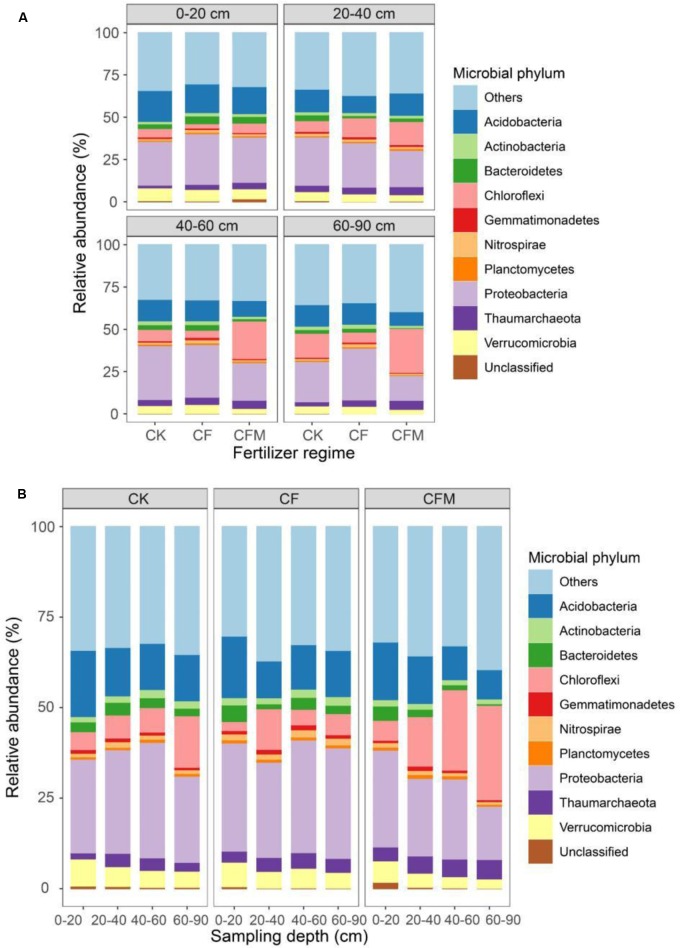
Relative abundances of the top 10 microbial phyla at different depths **(A)** under long-term fertilizer treatments **(B)**. ‘Others’ refer to those identified phyla beyond the top 10 phyla. CK, no fertilizer; CF, NPK fertilizer; CFM, NPK fertilizer combined with farmyard manure.

*Proteobacteria* and *Acidobacteria* were the most abundant phyla in the topsoil (0–20 cm), with relative abundances of 24–38% and 18–22%, respectively, and were followed by *Verrucomicrobia* (6.2–9.0%), *Chloroflexi* (2.0–7.2%), and *Bacteroidetes* (2.7–5.8%). Although the relative abundance of *Proteobacteria* showed a non-uniform variation pattern through the four different soil depths, the weighted mean values of the relative abundance were highest in the subsoil (20–90 cm) (**Figure 2**) (*P* = 0.05). The relative abundance of *Chloroflexi* increased significantly with increasing soil depth, while the relative abundances of *Acidobacteria*, *Verrucomicrobia*, and *Bacteroidetes* decreased (**Figure 2A**) (*P* = 0.05).

Compared to the control (CK), the relative abundance of *Proteobacteria* was higher in the CF and lower in the CFM treatment in topsoil (**Figure 2B**), and especially CF and also CFM resulted in higher relative abundances of *Betaproteobacteria* and *Gammaproteobacteria*, and lower relative abundances of *Deltaproteobacteria* in topsoil (Supplementary Figure S2) (*P* = 0.01). In addition, compared to the CK treatment, the relative abundance of *Alphaproteobacteria* was higher in the CF and lower in the CFM (Supplementary Figure S2). Finer taxonomic divisions also revealed the effect of fertilizer application in topsoil at the order level (**Table 2**). Compared to the CK, the relative abundance of *Nitrosomonadales* in *Betaproteobacteria* was 36 and 28 times higher in the CF and CFM treatments, respectively, and the abundance of *Methylophilales* was 7.3 and 2.9 times higher in the CF and CFM, respectively (*P* = 0.01). The abundances of *Xanthomonadales*, *Methylococcales*, and *Pseudomonadales* within *Gammaproteobacteria* were also higher in the CF and CFM treatments, whereas those of *Acidobacteria* were lower in the CF and CFM treatment (**Figure 2**). The abundances of minor phyla like *Nitrospirae* (Supplementary Figure S3) were higher in the fertilizer treatments whereas those of *Gemmatimonadetes* were lower (**Figure 2**) (*P* = 0.05).

**Table 2:** Relative abundances of selected orders within *Alphaproteobacteria*, *Betaproteobacteria*, *Deltaproteobacteria*, and *Gammaproteobacteria* at 0–20 and 20–90 cm depths under long-term fertilizer treatments.

Bacteria		Soil depth (cm)	Treatment		
		
Class	Order		CK	CF	CFM
Relative abundance (%)				
*Alphaproteobacteria*	*Sphingomonadales*	0–20	1.70 ± 0.17a^a^A^b^	1.94 ± 0.14aA	1.45 ± 0.02aA
		20–90	1.29 ± 0.02aB	1.31 ± 0.28aB	0.72 ± 0.16bB
	*Rhizobiales*	0–20	0.57 ± 0.02aA	0.66 ± 0.09aA	0.61 ± 0.05aA
		20–90	0.79 ± 0.05aA	0.97 ± 0.14aA	0.68 ± 0.02bA
*Betaproteobacteria*	*Nitrosomonadales*	0–20	0.00 ± 0.00bB	0.04 ± 0.01aA	0.03 ± 0.01aA
		20–90	0.02 ± 0.01aA	0.02 ± 0.01aB	0.01 ± 0.00aB
	*Methylophilales*	0–20	0.00 ± 0.00bB	0.03 ± 0.01aA	0.01 ± 0.00aA
		20–90	0.01 ± 0.00aA	0.01 ± 0.00aB	0.01 ± 0.00aA
	*Rhodocyclales*	0–20	1.52 ± 0.06aA	0.83 ± 0.11bA	0.79 ± 0.05bA
		20–90	0.47 ± 0.06aB	0.41 ± 0.09aB	0.30 ± 0.08aB
*Deltaproteobacteria*	*Myxococcales*	0–20	3.90 ± 0.46aA	3.86 ± 0.34aA	3.42 ± 0.03aA
		20–90	3.71 ± 0.25aA	4.36 ± 0.27aA	3.09 ± 0.23bA
	*Desulfuromonadales*	0–20	0.45 ± 0.06aB	0.65 ± 0.07aA	0.54 ± 0.05aA
		20–90	0.72 ± 0.01aA	0.72 ± 0.09aA	0.70 ± 0.10aA
*Gammaproteobacteria*	*Xanthomonadales*	0–20	1.37 ± 0.09bB	2.10 ± 0.33aA	1.95 ± 0.07aA
		20–90	2.81 ± 1.44aA	1.46 ± 0.40bB	1.52 ± 0.78bA
	*Methylococcales*	0–20	0.13 ± 0.02bA	0.34 ± 0.04aA	0.24 ± 0.02aA
		20–90	0.14 ± 0.01aA	0.12 ± 0.03aB	0.07 ± 0.03bB
	*Pseudomonadales*	0–20	0.08 ± 0.01bB	0.16 ± 0.02aB	0.12 ± 0.01aB
		20–90	0.71 ± 0.56aA	0.37 ± 0.17bA	0.48 ± 0.35bA


*Values at 20–90 cm depths are weighted means. CK, no fertilizer; CF, NPK fertilizer; CFM, NPK fertilizer combined with manure. Values in the column are mean ± SE (stand error). ^a^Treatment means within a depth followed by the same lower case letter (a, b, and c) are not significantly different (*P* > 0.05).*

*^b^Depth means within a column followed by the same upper case letter (A, B, and C) are not significantly different (*P* > 0.05).*

Long-term fertilizer application also changed the microbial community in subsoil (20–90 cm). For example, the relative abundance of *Gammaproteobacteria* was lower in the fertilizer treatments, especially in the CFM (Supplementary Figure S2). The relative abundances of *Alphaproteobacteria*, *Betaproteobacteria*, and *Gammaproteobacteria* were higher in the CF but lower in the CFM treatment compared to the CK (*P* = 0.05). For the finer taxonomic divisions, the relative abundances of the orders *Xanthomonadales*, *Methylococcales*, and *Pseudomonadales* within *Gammaproteobacteria* were lower in the fertilizer treatments. The relative abundances of orders *Sphingomonadales* and *Rhizobiales* within *Alphaproteobacteria*, and that of order *Myxococcales* within *Gammaproteobacteria* were higher in the CF but lower in the CFM treatment (**Table 2**) (*P* = 0.05). In addition, the abundances of phyla *Verrucomicrobia*, *Bacteroidetes*, and *Actinobacteria* (**Figure 2**) were lower in the two fertilizer treatments, and all of them were lowest in the CFM (*P* < 0.05). The relative abundances of *Gemmatimonadetes* and *Nitrospirae* were lower in the CFM and higher in the CF treatment (**Figure 2**) (*P* = 0.01).

### Community Structure, Variation, and Determinants

**FIGURE 3:**
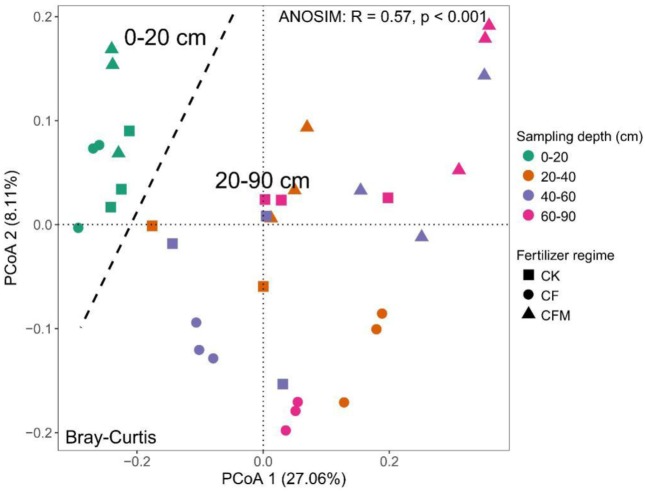
Principal coordinates analysis (PCoA) of the bacterial communities in the soil samples at 0–20 cm, 20–40 cm, 40-60 cm, and 60–90 cm depths under long-term fertilizer treatments. CK, no fertilizer; CF, NPK fertilizer; CFM, NPK fertilizer combined with farmyard manure.

**FIGURE 4:**
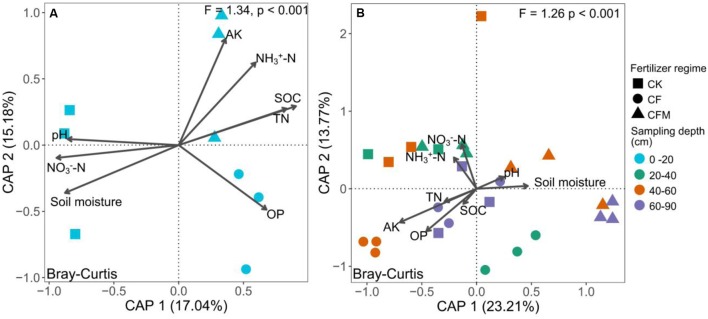
Distance based redundancy analysis (db-RDA, scaling = 2) based on Bray–Curtis dissimilarities of bacterial communities and measured soil properties at 0–20 cm **(A)** and 20–90 cm **(B)** depths under long-term fertilizer treatments. CK, no fertilizer; CF, NPK fertilizer; CFM, NPK fertilizer combined with farmyard manure. See **Figure 1** for the abbreviation of soil properties.

The topsoil samples were separated from subsoil samples in the principal coordinates analysis (PCoA) (**Figure 3**). The analysis of similarities (ANOSIM) further confirmed significant differences (*R* = 0.57, *P* < 0.001) between the topsoil and subsoil. Db-RDA that was used to quantify the impacts of edaphic factors on microbial community composition showed that SOC (*R*^2^ = 0.91, *P* = 0.006) was the major factor affecting variance in the bacterial community structure in the topsoil (**Figure 4A**). In addition to SOC, variance was also significantly linked to soil MO (*R*^2^ = 0.90, *P* = 0.006) and nitrate–N content (*R*^2^ = 0.90, *P* = 0.009). In the subsoil (**Figure 4B**), both soil OP and AK were strongly and significantly linked to community variance (OP: *R*^2^ = 0.45, *P* < 0.001; AK: *R*^2^ = 0.45, *P* < 0.001).

### Differential Abundance Analysis

**FIGURE 5:**
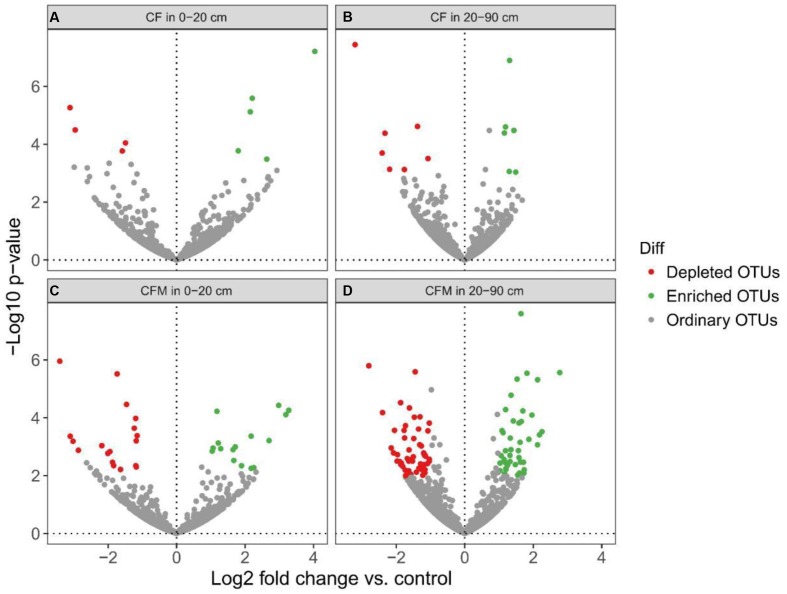
Volcano plots illustrating topsoil and subsoil OTUs that were significantly enriched (green) and depleted (red) by long-term fertilization compared with unfertilized control as determined by differential abundance analysis. Each point represents an individual OTU, and the *Y*-axis indicates the abundance fold change. **(A)** CF vs. control in the topsoil (0–20 cm); **(B)** CF vs. control in the subsoil (20–90 cm); **(C)** CFM vs. control in the topsoil (0–20 cm); **(D)** CFM vs. control in the subsoil (20–90 cm).

Differential abundance analysis was done to identify OTUs that were affected by fertilization (**Figure 5**). Enriched OTUs (eOTUs) and depleted OTUs (dOTUs) specifically represent OTUs more than two times higher or lower relative abundance (*P* < 0.05) in the long-term fertilization treatments. Altogether there were more OTUs enriched and depleted in CFM compared to CF in both the topsoil and subsoil. In the topsoil, there were 5 and 16 eOTUs, and 4 and 18 dOTUs in CF and CFM, respectively (**Figures 5A,C** and **Supplementary Data Sheet S1**). In the subsoil, 6 and 42 eOTUs, and 7 and 55 dOTUs were detected in CF and CFM, respectively (**Figures 5B,D** and **Supplementary Data Sheet S1**).

At the phylum level (Supplementary Figure S4A), the eOTUs in CF were mainly identified as *Proteobacteria*, *Bacteroidetes*, and *Gemmatimonadetes*, while those in CFM were *Proteobacteria*, *Acidobacteria*, *Bacteroidetes*, *Nitrospirae*, *Spirochaetes*, and *Thaumarchaeota* in the topsoil. The dOTUs in CF were mainly identified as *Acidobacteria*, *Proteobacteria* and *Verrucomicrobia*, while those in CFM were *Acidobacteria*, *Gemmatimonadetes*, *Proteobacteria*, and *Verrucomicrobia*. In the subsoil, the eOTUs in CF were mainly identified as *Acidobacteria*, *Actinobacteria*, *Proteobacteria*, and *Verrucomicrobia*, while those in CFM were *Chloroflexi*, *Acidobacteria*, *Actinobacteria*, *Proteobacteria*, and *Verrucomicrobia*. The dOTUs in CF were *Actinobacteria* and *Bacteroidetes*, while those in CFM were *Acidobacteria*, *Actinobacteria*, *Bacteroidetes*, *Proteobacteria*, and *Verrucomicrobia* (**Supplementary Data Sheet S1**).

At the genus level (Supplementary Figure S4B), the eOTUs in CF were identified as *Gemmatimonas* and *Bellilinea*, while those in CFM were *Nitrososphaera*, *Nitrospira*, *Dechloromonas*, *Gp4*, *Gp6*, *Gp10*, *Hydrogenophaga*, *Magnetospirillum*, *Bellilinea*, *Sulfuricurvum*, *Thiobacillus*, and *Treponema* in the topsoil. The dOTUs in CF were classified as Subdivision 3 *genera incertae sedis*, while those in CFM were *Gemmatimonas*, *Gp4*, *GP10*, *Gp21*, and Subdivision 3 *genera incertae sedis*. In the subsoil, the eOTUs in CF belonged to *Gp22* and Subdivision 3 *genera incertae sedis*, while those in CFM were Subdivision 3 *genera incertae sedis*, *Gp6*, *Gp9*, *Leptolinea*, and *Bellilinea*. The dOTUs in CF were unidentified, while those in CFM were *Gp3*, *Gp4*, *Gp10*, Subdivision 3 *genera incertae sedis*, *Sideroxydans*, *Steroidobacter*, *Phenylobacterium*, and *Geobacter* (**Supplementary Data Sheet S2**).

### Network Associations among Soil Microbial and Soil Properties

**FIGURE 6:**
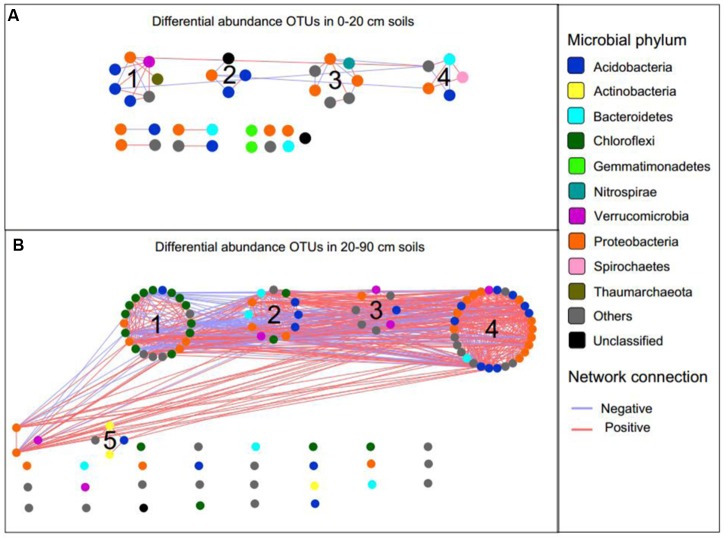
The correlation network of differentially abundant OTUs in topsoil **(A)** and subsoil **(B)**. Blue and red lines represent negative and positive correlation, respectively.

To explore the possible ecological interactions within members of microbial communities in top- and subsoil, we inferred two networks based on correlation between differentially abundant OTUs in topsoil and subsoil (**Figure 6**). The network of topsoil captured 37 potential associations among 38 OTUs (**Figure 6A**), while the network of OTUs in subsoil harbored 107 OTUs with 575 potential associations (**Figure 6B**). The basic network level topological features are listed in Supplementary Table S2. In the topsoil network, *Proteobacteria*, *Verrucomicrobia*, *Spirochaetes*, and *Nitrospirae* OTUs showed relatively high degree centrality (Supplementary Figures S5A,C), while several *Proteobacteria* and *Bacteroidetes* OTUs showed higher betweenness centrality compared to other OTUs (Supplementary Figures S5B,C). In the subsoil network, OTUs belonging to *Proteobacteria, Acidobacteria, Verrucomicrobia, Chloroflexi*, and *Bacteroidetes* showed higher degree centrality (Supplementary Figures S6A,C) while several *Proteobacteria*, *Chloroflexi, Bacteroidetes*, and *Verrucomicrobia* OTUs showed relatively higher betweenness centrality (Supplementary Figures S6B,C).

Using greedy modularity optimization algorithm, four and five network modules with more than four nodes were detected in the topsoil and subsoil networks, respectively. The topsoil network modules included *Nitrososphaera*, *Nitrospira*, *Hydrogenophaga*, *Thiobacillus*, *Sulfuricurvum*, and several *Acidobacteria* OTUs (**Supplementary Data Sheet S3**). The subsoil network module M1 contained *Leptolinea* and *Bellilinea* from phylum *Chloroflexi* and *Gp6* from phylum *Acidobacteria*. M2 contained genus *Gp4*, *Gp10* belonged to phylum *Chloroflexi*, *Steroidobacter* from *Proteobacteria*, and Subdivision 3 *genera incertae sedis* from *Verrucomicrobia*. M3 contained genus *Gp22* in *Acidobacteria*, Subdivision 3 *genera incertae sedis* from *Verrucomicrobia*. M4 contained *Sideroxydans* and *Phenylobacterium* from *Proteobacteria*, *Gp3* in *Acidobacteria*, Subdivision 3 *genera incertae sedis* from *Verrucomicrobia*. M5 was composed of only unidentified genus from *Micromonosporaceae* in *Actinobacteria* and *Holophagaceae* in *Acidobacteria* (**Supplementary Data Sheet S4**).

**FIGURE 7:**
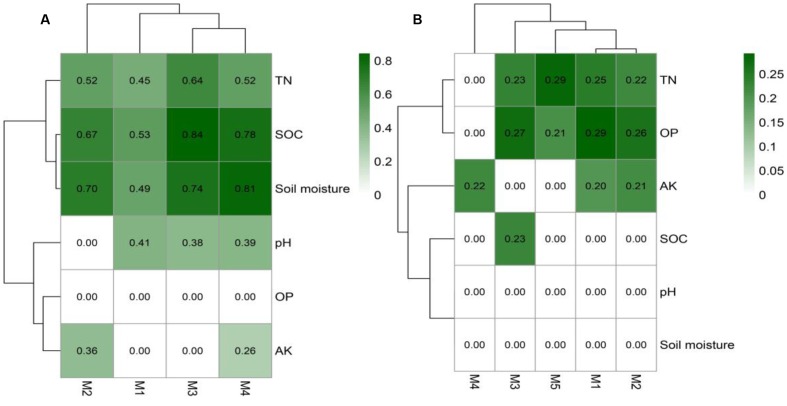
The correlation of modules in the topsoil **(A)** and subsoil **(B)** OTU networks using mantel test based on Bray-Curtis dissimilarity. See **Figure 1** for the abbreviation of soil properties.

The identification of node sources in each module indicated that almost all nodes in the topsoil network modules were OTUs differentially abundant in CFM (**Supplementary Data Sheet S3**). In the subsoil network, modules M1 and M2 consisted of OTUs differentially abundant in CFM, while M3, M4, and M5 included OTUs differentially abundant in CFM and CF (**Supplementary Data Sheet S4**). Mantel test based on Bray–Curtis dissimilarity indicated that the modules in topsoil network significantly (*p* < 0.05) correlated with multiple soil properties, of which SOC and soil moisture showed highest correlations (**Figure 7A**), suggesting that these two soil properties may be the primary forces in driving the abundance variation of these OTUs in CFM topsoil. In subsoil (**Supplementary Data Sheet S4**), the five modules showed different responses to environmental factors (**Figure 7B**). Modules M1 and M2 derived from CFM treatment showed higher correlations with TN and OP (**Figure 7B**). M3, M4, and M5 showed relatively higher correlations with OP, AK, and TN, respectively (**Figure 7B**).

## Discussion

### Effects of Depth and Long-Term Fertilization on Soil Physico-Chemical Parameters

We studied how fertilizer treatments affect soil bacterial and archaeal communities and soil properties. Soil physico-chemical parameters were strongly depth-dependent. Similar to earlier studies by Wang and Yang (2003) and Guo et al. (2010), lower pH in the fertilized topsoil might be due to that the fertilization stimulates nitrification and acidification as well as provides more organic acids to the soil. Higher SOC and TN concentrations in the topsoil may be explained by the fact that they are strongly related to root C inputs, and that manure and crop residues often accumulate in the topsoil. As in earlier studies, higher SOC in both CF and CFM treatments in the topsoil could result in higher soil moisture (Singh and Usha, 2003; Chen et al., 2007). Lower nitrate–N concentration in the subsoil may be due to less aerobic conditions, which result in higher losses of N through denitrification (Roldán et al., 2005), or there are more DNRA (dissimilatory nitrate reduction to ammonium) activity in the subsoil that produces ammonium that can be then assimilated by microorganisms and plants (Burger and Jackson, 2003). Though the application of P containing fertilizer increased the OP concentration, the OP concentration in the subsoil was lower than that in the topsoil which, probably due to the low solubility of phosphorus in slightly alkaline soil (El-Baruni and Olsen, 1979).

Soil properties change with agricultural management practices (Salako et al., 1999). Previous study reported that long-term CF treatments may acidify the soils, similar result was also observed in our study which showed that the soil pH was lowest in the CF soil (Guo et al., 2010). Manure containing fertilizers result in higher SOC content in the soil (Stark et al., 2007; Kuntal et al., 2008). In our study, SOC was higher in both the CF and CFM treatments compared to the control. Moreover, manure improves soil fertility since it contains more different nutrients than CFs (Fan et al., 2005). This was evident in our study where the concentrations of TN and AK were higher in the CFM treatment than in the CF treatment.

### Microbial Community Composition in Response to Long-Term Fertilization

Our differential abundance analysis revealed differences in microbial community structure between the CF and CFM treatments in both the topsoil and subsoil. This distinction between topsoil and subsoil should be associated with the direct or indirect effect of fertilization. As we hypothesized, more enriched OTUs belonging to *Nitrospirae*, *Spirochaetes*, *Acidobacteria*, and *Nitrososphaera* in the phylum *Thaumarchaeota* were detected in the soil treated with long-term CFM treatment compared to the CF treatment. *Nitrososphaera* is non-thermophilic Crenarchaeota and capable of oxidizing ammonia (Zhang and He, 2012). *Nitrospirae* played key roles in mediating the nitrite oxidation process in the greenhouse soil, and this result was thought to be attributed to the high substrate affinity of *Nitrospirae* (Freitag et al., 2005; Xia et al., 2010). As *Nitrospirae* phylum is involved in nitrite oxidation (Fierer et al., 2007; Lücker et al., 2010), the enrichment of *Nitrospirae* OTUs in the CFM soil may have had positive effects on nitrification.

The 32 years of CF and CFM fertilizer amendment changed the relative abundances of nitrifying archaea and bacteria in the topsoil and even the subsoil, and there were distinct differences in communities between the CF and CFM treatments. Application of urea fertilizer could lead to the enrichment of NH_4_^+^–N in the soil due to fast hydrolysis of urea-N, which then leaks downward during irrigation and is gradually re-absorbed in deeper soil layers (Chien et al., 2009). This may favor the growth of ammonia-oxidizing microbes under long-term fertilizer treatments. Our study showed that, comparing to CK, the two fertilizer treatments (CF and CFM) decreased both ammonium–N content and nitrate–N content within the whole profile (**Table 1**), which also indicated the increased activity of ammonia and nitrite oxidizers during fertilizer application. Ammonia and nitrite oxidizers are critical to soil N cycling (Fierer et al., 2007), and the application N containing fertilizer in the current study was likely to result in different composition and structure of these communities.

### Changes in Taxa Abundances with Soil Depth

The subsoil microbial communities were distinct in composition and structure from topsoil communities (Fierer et al., 2003a,b). Based on our differential abundance analysis, primarily OTUs from *Proteobacteria*, *Acidobacteria*, and *Gemmatimonadetes* were enriched in the topsoil, while *Chloroflexi* and *Actinobacteria* were enriched in the subsoil. *Betaproteobacteria* is considered as copiotrophic bacteria that flourish in soils with enriched nutrients (Fierer et al., 2007), which probably explains why mostly *Betaproteobacteria* OTUs were enriched in the topsoil. *Acidobacteria* include many oligotrophic members (Nemergut et al., 2010; Pascault et al., 2013), yet *Acidobacteria* like *Gp4* and *Gp6* were abundant in soils with higher contents of soil C whereas *Gp1* and *Gp7* were not (Liu et al., 2014; Li et al., 2017). Similarly, in our study both enriched and depleted *Acidobacteria* were detected in the soil profile, and the relative abundance of *Acidobacteria* was higher in the topsoil than in the subsoil. *Chloroflexi* are facultative anaerobic and have a recognized role as heterotrophic oligotrophs in soils, having the ability to survive on recalcitrant plant polymers (Yabe et al., 2010; Hug et al., 2013; King, 2014). The relative abundance of *Chloroflexi* was relatively lower compared to other studies (Zhao et al., 2014; Chen et al., 2016), but similar to the levels reported by Li et al. (2014). Their enrichment in the subsoil confirmed their adaptation to growth under nutrient limitation (Engel et al., 2010; Hug et al., 2013; Barton et al., 2014).

At the genus level the most enriched OTUs in the topsoil were *Ohtaekwangia*, *Gemmatimonas*, and *Nitrospira*. Members of the *Ohtaekwangia* genus, potential petroleum hydrocarbon degraders, were widely distributed in plant rhizosphere (Zhang et al., 2011; McGenity et al., 2012; Tejeda-Agredano et al., 2013; Gaggìa et al., 2013). *Ohtaekwangia* may contribute to the transformation of soil carbon derived from the plant (Naumoff and Dedysh, 2012), possibly explaining the increase of their relative abundance. *Gemmatimonas* species were able to modulate carbon and nitrogen intake according to their metabolic needs under various conditions and were abundant in soils amended with pyrogenic organic material (Carbonetto et al., 2014; Xu et al., 2014; Whitman et al., 2016), indicating that they are likely to decompose polyaromatic carbon compounds. *Gemmatimonas* species accumulated polyphosphate and were stimulated by P fertilizer (Zhang et al., 2003; Su et al., 2015). Members of *Nitrospira* are mostly uncultured nitrite-oxidizing bacteria (Palomo et al., 2016). In this study, *Nitrospira* OTUs were more relevant to the CFM than to the CF treatment in the topsoil, probably due to the lower C/N ratio of manure, which mobilizes soil N, increases its mineralization and the availability of mineral nitrogen (Leite et al., 2017).

The most enriched OTUs in the subsoil were genus Subdivision 3 *genera incertae sedis, Leptolinea, Bellilinea*, and some subgroups of *Acidobacteria*. Subdivision 3 *genera incertae sedis* is affiliated to the phylum *Verrucomicrobia*, which was negatively correlated with soil fertility and increased in abundance with increasing available N, P, K, and SOC obtained from cotton straw (Huang et al., 2012; Navarrete et al., 2015). Genus *Leptolinea* and *Bellilinea* belong to class *Anaerolineae* in the oligotrophic *Chloroflexi* subphylum I, and needed to associate with other microbes to grow efficiently (Narihiro et al., 2012). Taken together, our results showed that specific microbial taxa involved in decomposition of organic compounds and in C, N, and P transformations were substantially enriched or depleted in the top- and subsoil by long-term fertilization.

### OTU Association Networks in Top- and Subsoil

Since C and N are essential nutrients for microbial growth, soil C and N are expected to show strong associations with taxa affected directly or indirectly by long-term fertilization in different soil depths. Our co-occurrence based network analysis revealed that in the topsoil SOC and soil moisture had strong positive correlations with microbial taxa, e.g., genus *Nitrososphaera* from phylum *Thaumarchaeota*, *Nitrospira* of *Nitrospirae*, *Hydrogenophaga*, and *Thiobacillus and Sulfuricurvum* from *Proteobacteria*, and several subgroups of *Acidobacteria*. In the subsoil soil OP and TN showed strong positive correlations with different genera belonging to phyla *Chloroflexi*, *Acidobacteria*, *Proteobacteria*, and *Verrucomicrobia* that participate in soil nutrient transformations as discussed above. Betweenness centrality is linked to the importance of the control potential an OTU exerts over the interactions of other OTUs in that network. OTUs with high betweenness centrality values might possess high impact on other interactions in the community (Greenblum et al., 2012). Keystone nodes in co-occurrence networks tend to have maximum betweenness centrality values (Vick-Majors et al., 2015; Banerjee et al., 2016). Based on betweenness centrality, most keystone bacterial nodes in our study belonged to the most abundant phyla *Proteobacteria* and *Bacteroidetes* in both top- and subsoil. Within the *Proteobacteria*, *Hydrogenophaga* was identified as the keystone species in the topsoil, while genus *Phenylobacterium* and *Steroidobacter* were identified as keystones in the subsoil. These genera have positive effects on soil nutrient cycling and have been reported as beneficial microorganisms in soil. Genus *Hydrogenophaga* belongs to order *Burkholderiales*, members of which have shown ability to decompose degrade high molecular weight organic compounds and utilize sulfanilic acid as the sole carbon and energy source (Ding et al., 2012). Genus *Phenylobacterium* and *Steroidobacter* belong to *Caulobacterales* and *Xanthomonadales*, respectively. *Phenylobacterium* species were reported to be favored by a compound in the organic material applied or metabolites available at a specific stage in the degradation process (Cruz-Barrón et al., 2017). Genus *Steroidobacter* in SOC-poor soil could be enriched by the application of soybean residues and was thought to be associated with C and N cycling as this genus utilizes only a narrow range of organic substrates with nitrate as the electron acceptor (Lian et al., 2017). Similarly, the genus *Steroidobacter* was found to be one keystone in the subsoil in this study. As the concentration of soil total nitrogen and SOC in the subsoil was relative lower than those in the topsoil (**Figure 1**), *Steroidobacter* may contribute to the SOC decomposition for N demand in nutrient poor soil (Lian et al., 2017).

In summary, this study demonstrated that the 32-year long fertilization treatments not only affected the soil physical–chemical parameters but also the composition of the bacterial and archaeal communities both in the top- and subsoil. Our previous study revealed that yields of rice and wheat were comparable and occasionally higher under CFM than under CF treatment. *Proteobacteria* and *Acidobacteria* were the most abundant phyla in topsoil. Compared to CF treatment, different bacterial taxa were enriched under CFM treatment in both topsoil and subsoil. These taxa are widely distributed in soil and involved in soil nutrient transformations. Taken together, the spatial variability of soil properties due to long-term fertilization strongly shaped the bacterial and archaeal community composition and their interactions at both high and low taxonomic levels and resulted in distinct association patterns among the specific taxa in topsoil and subsoil.

## Author Contributions

YG, ST, and XZ designed the study. YG, QX, and KZ analyzed the data and wrote the manuscript. SL, XY, and LZ collected and analyzed soil samples. ST and QC managed the long-term field experiment. All authors reviewed the manuscript.

## Conflict of Interest Statement

The authors declare that the research was conducted in the absence of any commercial or financial relationships that could be construed as a potential conflict of interest.

## Footnotes

1 https://www.ncbi.nlm.nih.gov/sra/

## Supplementary Material

**DATA SHEET S1 |** All the significantly differential OTUs at the phylum level within the whole soil profile.

**DATA SHEET S2 |** All the significantly differential OTUs at the genus level within the whole soil profile.

**DATA SHEET S3 |** Differential type of network nodes in the topsoil (0–20 cm).

**DATA SHEET S4 |** Differential type of network nodes in the subsoil (20–90 cm).
