# Supplementary material for: Long-term Fertilization Structures Bacterial and Archaeal Communities along Soil Depth Gradient in a Paddy Soil
Source: Front Microbiol. 2017 Aug 15;8:1516. doi: 10.3389/fmicb.2017.01516 (PMC5559540; doi:10.3389/fmicb.2017.01516)
Supplement: Supplementary file 5 [file Table_1.docx]

**Table S1.** Statistical indices indicating the percent coverage of the 36 soil samples within the whole profile (0-90 cm) under different long-term fertilizer treatments.

| Soil depth | Treatment^a^ | | |
| --- | --- | --- | --- |
|  | CK | CF | CFM |
| 0-20 cm | 0.796±0.017^b^ | 0.778±0.008 | 0.802±0.004 |
| 20-40 cm | 0.787±0.009 | 0.814±0.002 | 0.786±0.009 |
| 40-60 cm | 0.782±0.020 | 0.795±0.019 | 0.819±0.031 |
| 60-90 cm | 0.824±0.008 | 0.789±0.024 | 0.857±0.015 |

^a^ The three different long term fertilization treatments. CK: no fertilizer; CF: NPK fertilizer; CFM: NPK fertilizer combined with farmyard manure. ^b^ Good's coverage at a distance of 0.03. Values in the column are mean ± SE (stand error).
