# Supplementary material for: Long-term Fertilization Structures Bacterial and Archaeal Communities along Soil Depth Gradient in a Paddy Soil
Source: Front Microbiol. 2017 Aug 15;8:1516. doi: 10.3389/fmicb.2017.01516 (PMC5559540; doi:10.3389/fmicb.2017.01516)
Supplement: Supplementary file 6 [file Table_2.docx]

**Table S2.** The network-level topological features of correlation network of differentially abundant OTUs in the topsoil and subsoil.

| Sampling depth (cm) | Node counts | Edge counts | Average degree | Average path length | Clustering coefficient | Module counts | Modularity | Network diameter | Degree assortativity | Network density |
| --- | --- | --- | --- | --- | --- | --- | --- | --- | --- | --- |
| Topsoil (0-20) | 38 | 37 | 1.95 | 3.49 | 0.42 | 15 | 0.67 | 7 | 0.14 | 0.05 |
| Subsoil (20-90) | 107 | 575 | 10.75 | 2.56 | 0.62 | 34 | 0.18 | 7 | 0.11 | 0.10 |
