# Supplementary material for: Long-term Fertilization Structures Bacterial and Archaeal Communities along Soil Depth Gradient in a Paddy Soil
Source: Front Microbiol. 2017 Aug 15;8:1516. doi: 10.3389/fmicb.2017.01516 (PMC5559540; doi:10.3389/fmicb.2017.01516)
Supplement: Supplementary file 7 [file Image_1.pdf]

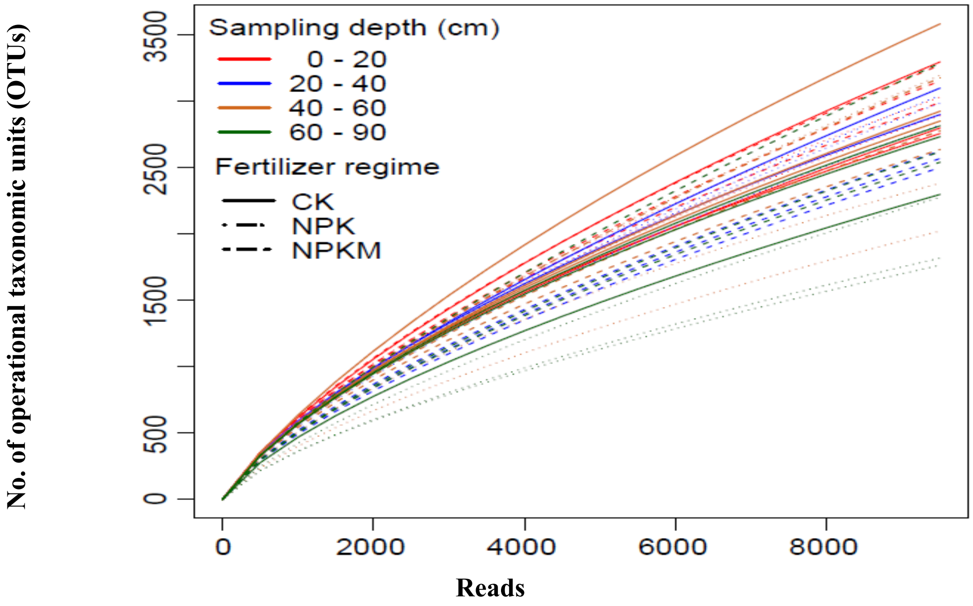

Figure S1. Rarefaction curves (cutoff 0.03) of the soil microbiota within the whole soil profile (0-90 cm) under different long-term fertilizer treatments.
