# Supplementary material for: Long-term Fertilization Structures Bacterial and Archaeal Communities along Soil Depth Gradient in a Paddy Soil
Source: Front Microbiol. 2017 Aug 15;8:1516. doi: 10.3389/fmicb.2017.01516 (PMC5559540; doi:10.3389/fmicb.2017.01516)
Supplement: Supplementary file 8 [file Image_2.pdf]

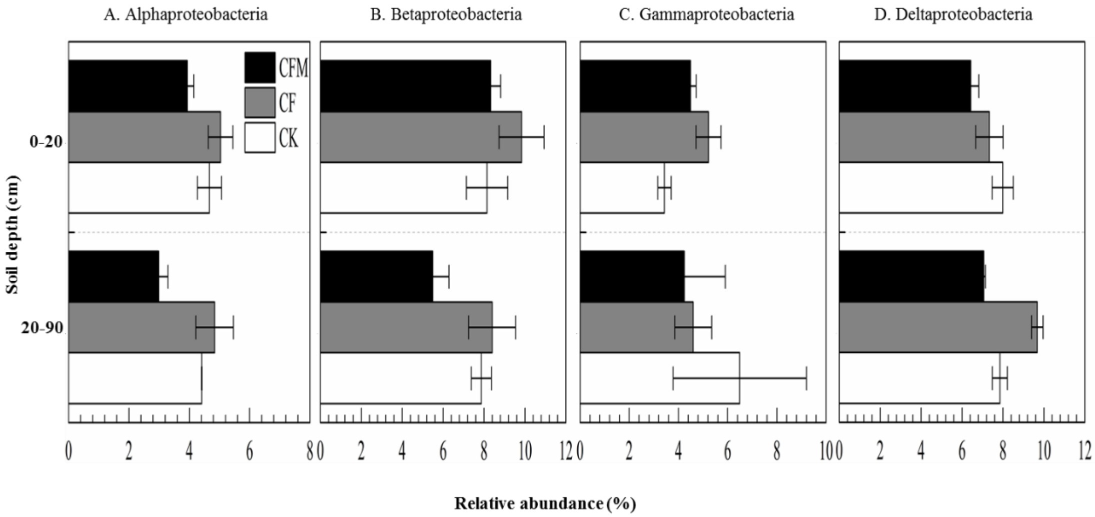

Figure S2. Relative abundances of *Alphaproteobacteria* (A), *Betaproteobacteria* (B), *Gammaproteobacteria* (C) and *Deltaproteobacteria* (D) at 0-20 and 20-90 cm depths under different long-term fertilizer treatments. Values at 20-90 cm depths are weighted means. CK: no fertilizer; CF: NPK fertilizer; CFM: NPK fertilizer combined with farmyard manure. Error bars represent the standard error of the mean.
