# Supplementary material for: Long-term Fertilization Structures Bacterial and Archaeal Communities along Soil Depth Gradient in a Paddy Soil
Source: Front Microbiol. 2017 Aug 15;8:1516. doi: 10.3389/fmicb.2017.01516 (PMC5559540; doi:10.3389/fmicb.2017.01516)
Supplement: Supplementary file 9 [file Image_3.pdf]

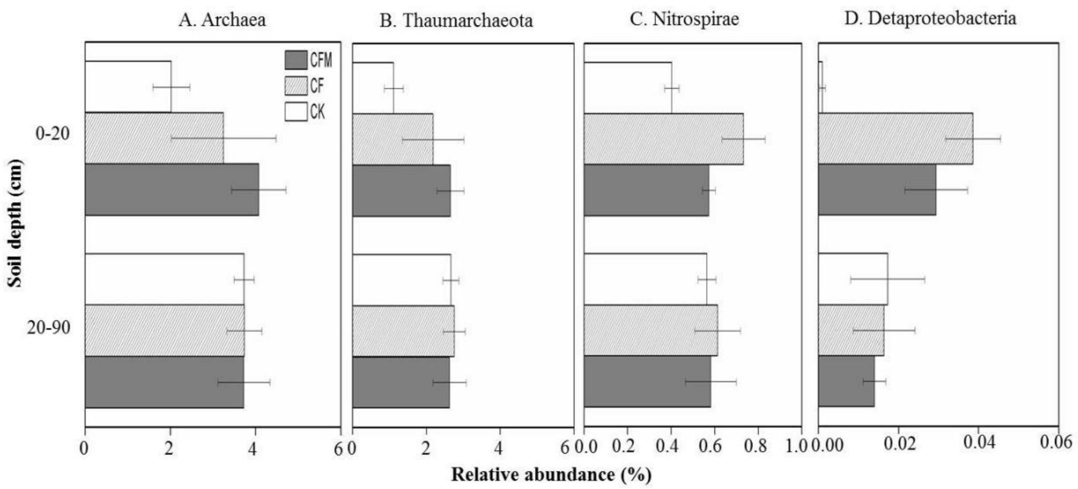

Figure S3. Relative abundances of *Archaea* (A), *Thaumarchaeota* (B), *Nitrospirae* (C) and *Nitrosomonadales* (D) at 0-20 and 20-90 cm depths under different long term fertilizer treatments. Values at 20-90 cm depths are weighted means. CK: no fertilizer; CF: NPK fertilizer; CFM: NPK fertilizer combined with farmyard manure. Error bars represent the standard error of the mean.
