# Supplementary figures and images for: Long-term Fertilization Structures Bacterial and Archaeal Communities along Soil Depth Gradient in a Paddy Soil
Source: Front Microbiol. 2017 Aug 15;8:1516. doi: 10.3389/fmicb.2017.01516 (PMC5559540; doi:10.3389/fmicb.2017.01516)

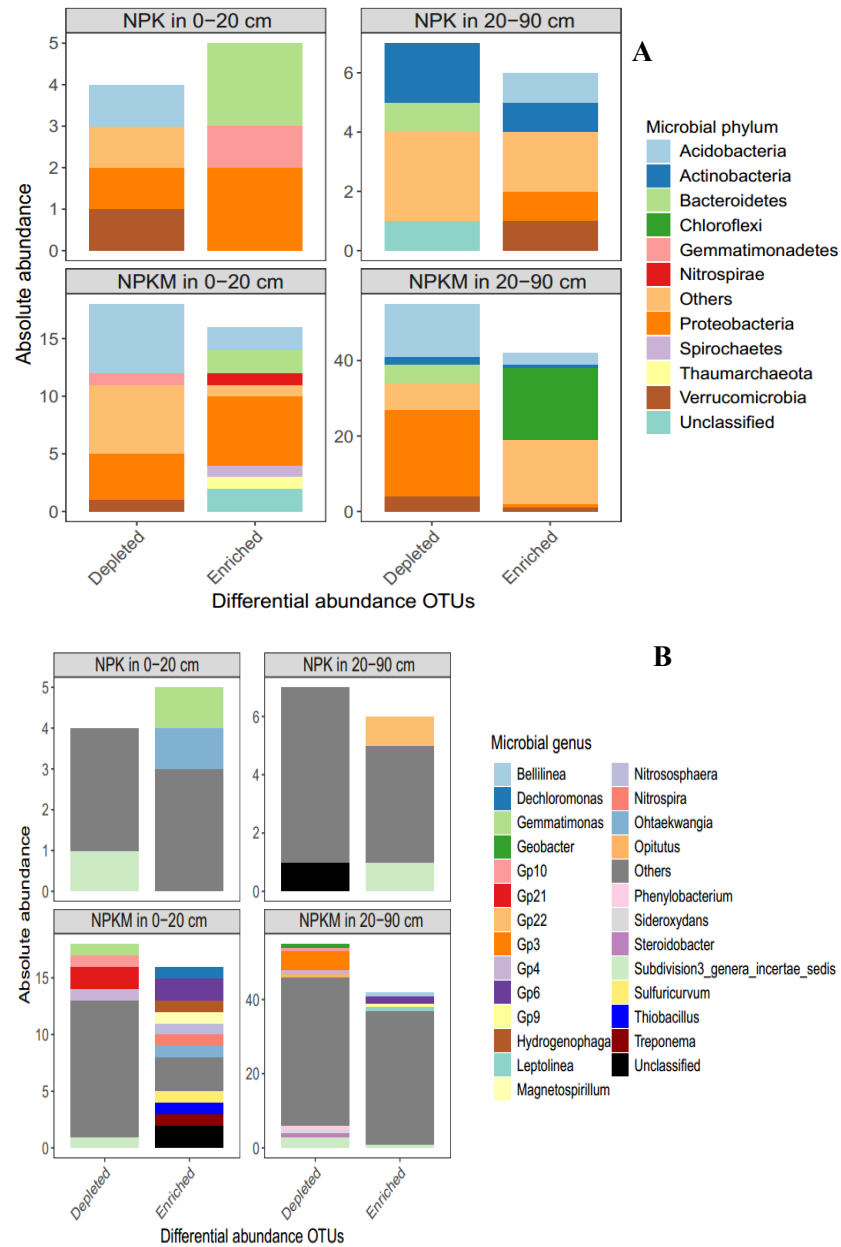

Supplement: Supplementary file 10 [file Image_4.pdf]

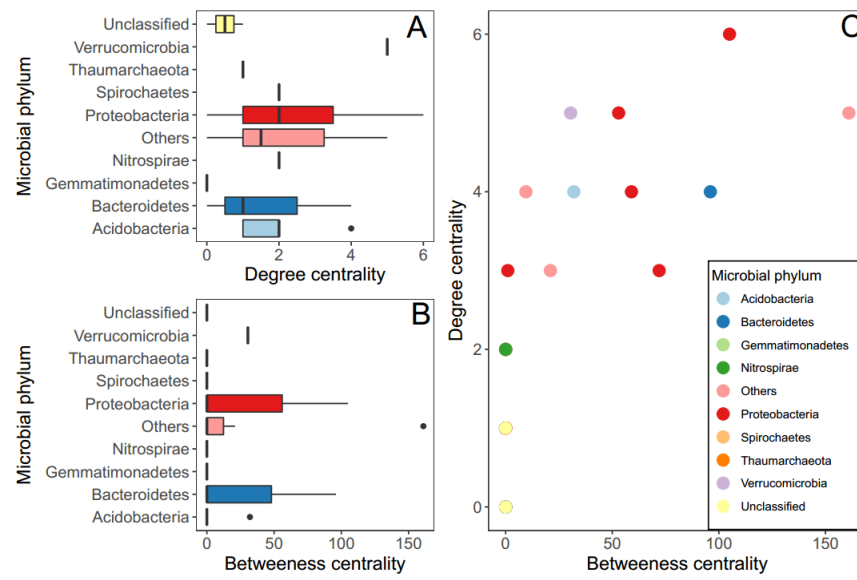

**Figure S5.** The degree and betweenness centrality of correlation network of the topsoil (0-20 cm).

Supplement: Supplementary file 11 [file Image_5.pdf]

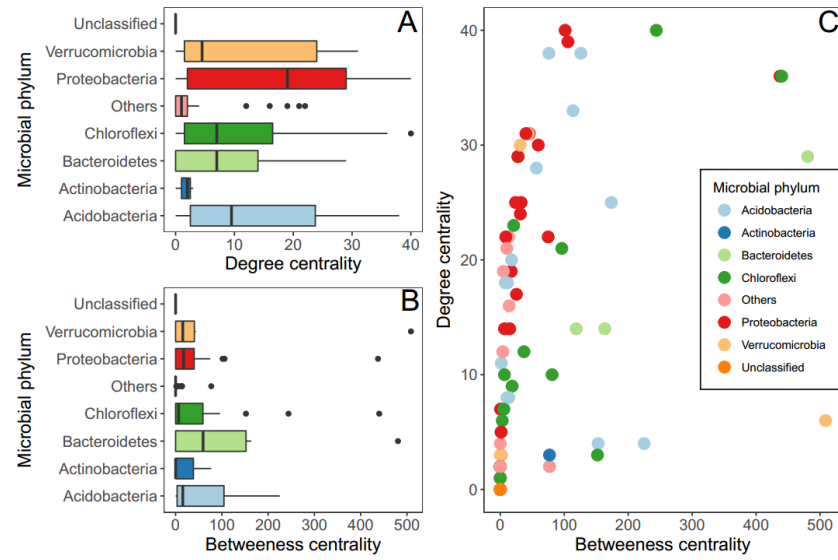

**Figure S6.** The degree and betweenness centrality of correlation network of subsoils (20-90 cm).

Supplement: Supplementary file 12 [file Image_6.pdf]
